# Vertical and Horizontal Ridge Augmentation with Titanium-Reinforced Dense PTFE and Reinforced PTFE Mesh: A Prospective Comparative Case Series

**DOI:** 10.3390/jfb17050234

**Published:** 2026-05-07

**Authors:** Liliana Andrea Silva, Pedro Sousa Gomes, Maria Helena Fernandes, Marta García-García, Octavi Camps-Font

**Affiliations:** 1 Cirurgia Oral, Faculdade de Medicina Dentária, Universidade do Porto, 4200-393 Porto, Portugal; 2 Bone Lab, Faculdade de Medicina Dentária, Universidade do Porto, 4200-393 Porto, Portugal; mhfernandes@fmd.up.pt; 3 LAQV/REQUIMTE, Faculdade de Medicina Dentária, Universidade do Porto, 4200-393 Porto, Portugal; 4 Faculty of Medicine and Health Sciences, Universitat de Barcelona, 08036 Barcelona, Spain; martagarciagarcia@ub.edu (M.G.-G.); ocamps@ub.edu (O.C.-F.); 5 IDIBELL Research Institute, 08908 Barcelona, Spain

**Keywords:** TiR-dPTFE membrane, RPM membrane, bone augmentation, vertical bone deficiencies, vertical bone gain, GBR, case series

## Abstract

Objectives: This study aimed to compare vertical bone gain (VBG) and horizontal bone gain (HBG) after guided bone regeneration using titanium-reinforced dense PTFE (TiR-dPTFE) versus reinforced PTFE mesh (RPM) at 9 and 12 months on three-dimensional tomographic imaging, and to perform histological assessment in selected cases. Materials and Methods: This prospective comparative case series included 14 patients (46 vertical ridge defect sites) treated with guided bone regeneration using either Ti-reinforced dPTFE membranes (TiR-dPTFE; n = 23) or resorbable porcine collagen membranes (RPM; n = 23). All sites received a 60:40 mixture of autogenous bone chips and anorganic bovine bone mineral (ABBM). After 9 months, during implant placement, a protective secondary augmentation using a 70:30 ABBM/autogenous mixture was performed and covered with a collagen membrane. Vertical and horizontal bone gain (VBG, HBG) were assessed on standardized matched CBCT cross-sections obtained at 9 and 12 months. Core biopsies were harvested at implant placement (9 months) for histological evaluation. Surgical and healing complications were recorded. Results: Both membranes produced significant VBG. TiR-dPTFE achieved greater VBG than RPM at 9 months (*p* = 0.045) and 12 months (*p* = 0.012) and remained stable from 9 to 12 months, whereas RPM showed a significant decline over time (MDa −0.48 mm; 95% CI −0.64 to −0.31; *p* < 0.001). HBG was similar between groups at both time points (*p* = 0.918 and *p* = 0.922). No major clinical complications occurred. Histology at 9 months confirmed vital bone formation and graft integration in both groups. Conclusions: Both TiR-dPTFE and RPM are reliable options for vertical ridge augmentation; TiR-dPTFE yielded superior and more stable vertical gains over 12 months, with comparable horizontal outcomes. Clinical Relevance: TiR-dPTFE may offer enhanced vertical augmentation, while both membranes remain suitable for implant site development.

## 1. Introduction

Guided bone regeneration (GBR) is a well-established surgical approach for reconstructing alveolar bone defects, enabling both vertical and horizontal augmentation to support optimal implant placement and long-term stability [1,2,3]. Its predictability relies on the use of a barrier membrane that creates a secluded environment conducive to osteogenic cell proliferation while excluding soft-tissue invasion [1].

Barrier membranes can be broadly categorized as resorbable or non-resorbable, each presenting specific biological and clinical characteristics [4,5]. Resorbable membranes, often collagen-based, have the advantage of avoiding a second surgery for removal but may exhibit limited form stability, unpredictable degradation rates, and insufficient space maintenance during healing [4,5]. In contrast, non-resorbable PTFE membranes remain the gold standard where space maintenance is critical [6]. Expanded PTFE (ePTFE) and dense PTFE (dPTFE) differ mainly in pore size: the smaller pores of dPTFE (<3 μm) limit bacterial penetration and facilitate easier removal [7,8].

Titanium reinforcement further improves the mechanical stability of dPTFE membranes, making them particularly suitable for challenging vertical ridge augmentation procedures [9]. Clinical studies have consistently demonstrated substantial vertical bone gains using titanium-reinforced dPTFE (TiR-dPTFE) combined with autogenous bone and xenograft mixtures [9,10]. Similarly, titanium mesh (Ti-mesh) devices provide greater rigidity and superior three-dimensional stability but may increase the risk of soft-tissue exposure and complicate removal due to soft-tissue ingrowth [2,11,12,13,14], and their regenerative performance may also be influenced by mesh porosity, with smaller pore sizes having been associated with improved bone regeneration outcomes [13].

To address the limitations associated with conventional titanium-reinforced non-resorbable barriers, hybrid devices such as reinforced PTFE mesh membranes (RPM) have been introduced, combining a dense PTFE (dPTFE) occlusive barrier with an embedded titanium framework and calibrated microporosities [12]. This configuration is intended to maintain the space-preserving function required for vertical ridge augmentation while promoting improved biological interaction with the overlying periosteum through controlled vascular access. The presence of perforations has been proposed to partially reconcile the need for barrier occlusivity with the potential benefits of periosteal-derived vascular supply, which may support graft remodeling and maturation without compromising soft-tissue exclusion [14]. Preliminary experimental and clinical observations suggest that this design may contribute to enhanced graft stability and potentially reduced membrane exposure rates compared with conventional titanium-reinforced PTFE barriers [12]; however, direct comparative clinical evidence supporting these effects in vertical ridge augmentation procedures remains limited.

Therefore, this prospective case series aimed to compare the clinical and radiographic performance of TiR-dPTFE and RPM membranes in vertical ridge augmentation procedures. The primary outcome was bone regeneration, quantified as vertical and horizontal dimensional gain, while secondary outcomes included healing events and the histological characteristics of the regenerated tissue.

Within this design, the working hypothesis posited that the macroporous design of the RPM mesh, by enhancing periosteal vascularization and biological integration, would result in bone regeneration outcomes comparable to or superior to those achieved with the conventional TiR-dPTFE membrane, without increasing the risk of clinical complications.

## 2. Materials and Methods

### 2.1. Study Design

This prospective comparative case series was conducted collaboratively between the Faculty of Dental Medicine, University of Porto (Porto, Portugal), and a private clinical center (São João da Madeira, Portugal); all surgical procedures were performed by a single experienced surgeon (L.S.) at the private clinic, following an identical clinical and analytical protocol. The study protocol was approved by the Ethics Committee for Health at the Faculty of Dental Medicine, University of Porto (protocol code: 2022/05-FMDUP), and complied with the principles of the Declaration of Helsinki for research involving human participants [15]. The study is reported in accordance with the STROBE recommendations for observational studies [16]. All participants received a detailed explanation of the study objectives, procedures, and potential risks, and provided written informed consent prior to enrolment.

### 2.2. Study Population

Consecutive patients presenting with partial (>2 teeth missing) or total edentulism in the maxilla or mandible and requiring three-dimensional bone augmentation for prosthetically guided implant placement were prospectively enrolled between January 2022 and June 2024, according to predefined inclusion and exclusion criteria. Eligible participants were recruited sequentially during routine clinical care at the participating center, ensuring that no patient meeting the criteria was omitted or selectively included. The inclusion criteria were: (1) male or female patients aged ≥18 years, (2) alveolar ridge deficiency sites necessitating bone augmentation prior to implant placement, (3) a vertical bone defect > 3 mm, and (4) a full-mouth O’Leary plaque score and full-mouth bleeding on probing score <10%.

In addition to the general contraindications for implant surgery, the following exclusion criteria were applied: (1) a history of local radiotherapy in the head and neck region, (2) smoking, (3) alcoholism or substance abuse, (4) untreated or active periodontitis, (5) pregnancy or breastfeeding, (6) participation in a randomized controlled trial within the past 30 days, and (7) any conditions or circumstances that, in the investigator’s opinion, could prevent the completion of study participation or interfere with the analysis of study results, such as a history of noncompliance or unreliability. The investigational devices consisted of two commercially available non-resorbable barrier materials for GBR: a reinforced PTFE mesh (RPM, Cytoplast^®^, Osteogenic Biomedical, Lubbock, TX, USA) and a titanium-reinforced dense PTFE membrane (TiR-dPTFE, Cytoplast^®^, Osteogenic Biomedical). Patients were consecutively enrolled and assigned to either the TiR-dPTFE or RPM group according to a predefined clinical allocation sequence determined by the treating surgeon within a standardized treatment protocol.

A flowchart summarizing patient screening, eligibility assessment, group allocation, follow-up, and inclusion in the final analysis is provided in Supplementary Figure S1.

### 2.3. Sample Size Calculation

The present investigation was conceived as a prospective exploratory trial; therefore, the sample-size estimation was not intended to support confirmatory or hypothesis-testing purposes but rather to ensure that the study included a minimally adequate number of observations to explore potential differences between membranes. A preliminary sample-size calculation was performed using G*Power v.3.1.3 software (Heinrich-Heine Universität, Düsseldorf, Germany) to approximate the number of implant sites required to detect a clinically relevant difference in vertical bone gain (VBG). Under the assumption that a difference of at least 3 mm would be of clinical relevance, with a common standard deviation (SD) of 2 mm [11], an allocation ratio of 1:1, a significance level of 0.05, a statistical power of 80%, and allowing for a 10% exclusion rate, an initial estimate of 10 independent implant sites (5 sites per membrane group) was obtained. However, because the study involved a hierarchical structure (multiple implants nested within individual patients), this preliminary estimate was adjusted to account for intrasubject correlation. Assuming a moderate intraclass correlation of 0.5 and an average of three implant sites per patient, the required number increased to approximately 20 implant sites, which corresponded to an estimated 7 participants per group.

### 2.4. Interventions

#### 2.4.1. Preoperative Procedures

**Figure 1:**
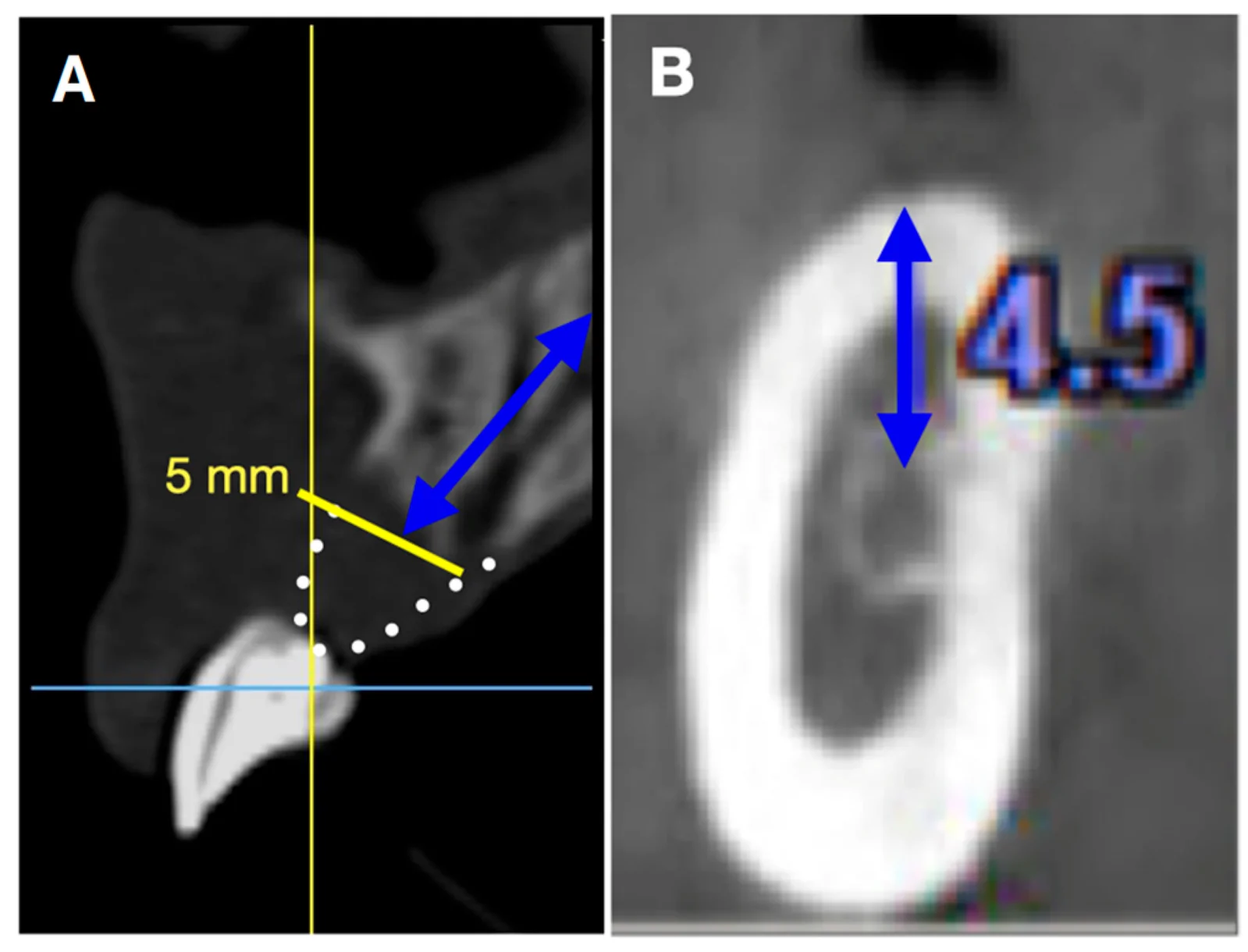
Cone-beam computed tomography (CBCT) reference methodology used for baseline vertical bone defect (VBD) measurements (T0). (**A**) Measurement of anterior maxillary vertical defect height, defined as the distance from the nasal floor to the viable edge of the residual alveolar crest. (**B**) Measurement of mandibular vertical defect height, defined as the distance from the viable edge of the residual alveolar crest to the superior border of the inferior alveolar nerve canal.

All participants received personalized professional oral hygiene instructions, along with comprehensive full mouth supragingival scaling and polishing. For each patient, a cone-beam computed tomography (CBCT) (Newtom Giano^®^, Verona, Italy; 0.5 mm/voxel, 90 Kv, 37.07 mA, 18 s) was made (T0). The Digital Imaging and Communication in Medicine (DICOM) files obtained from the CBCT were imported to DTX Studio Implant^®^ software, version 3.6 (Nobel Biocare AB, Gothenburg, Sweden). The virtual bone augmentation process was conducted by a single blinded clinician (F.F.) with expertise in digital implant planning. The amount of vertical and horizontal bone was measured before bone-grafting. For residual bone height, measurements were taken from the viable edge of the alveolar crest to the anatomical limits [17,18]. For residual bone width, a perpendicular line was drawn following residual bone inclination, and subsequently a horizontal line recorded bone width at 5 mm from the crest [18,19] (Figure 1).

#### 2.4.2. Surgical Protocol

All surgical procedures were performed under intravenous sedation by a single experienced surgeon (L.S.), following the protocol of Tinti & Parma-Benfenati [20], later modified by Urban [21,22]. A mid-crestal horizontal incision was made in the mandible and a para-crestal incision in the maxilla, complemented by two buccal vertical releasing incisions and an oblique lingual mesial incision. A full-thickness mucoperiosteal flap was carefully elevated, with distinct mobilization techniques for each aspect: on the lingual side, elevation extended to the mylohyoid line with detachment of the mylohyoid muscle insertion; on the buccal side, mobilization was achieved via a longitudinal periosteal incision in the vestibular fornix and partial-thickness dissection. Cortical perforations were created to enhance vascularization [22,23,24].

**Figure 2:**
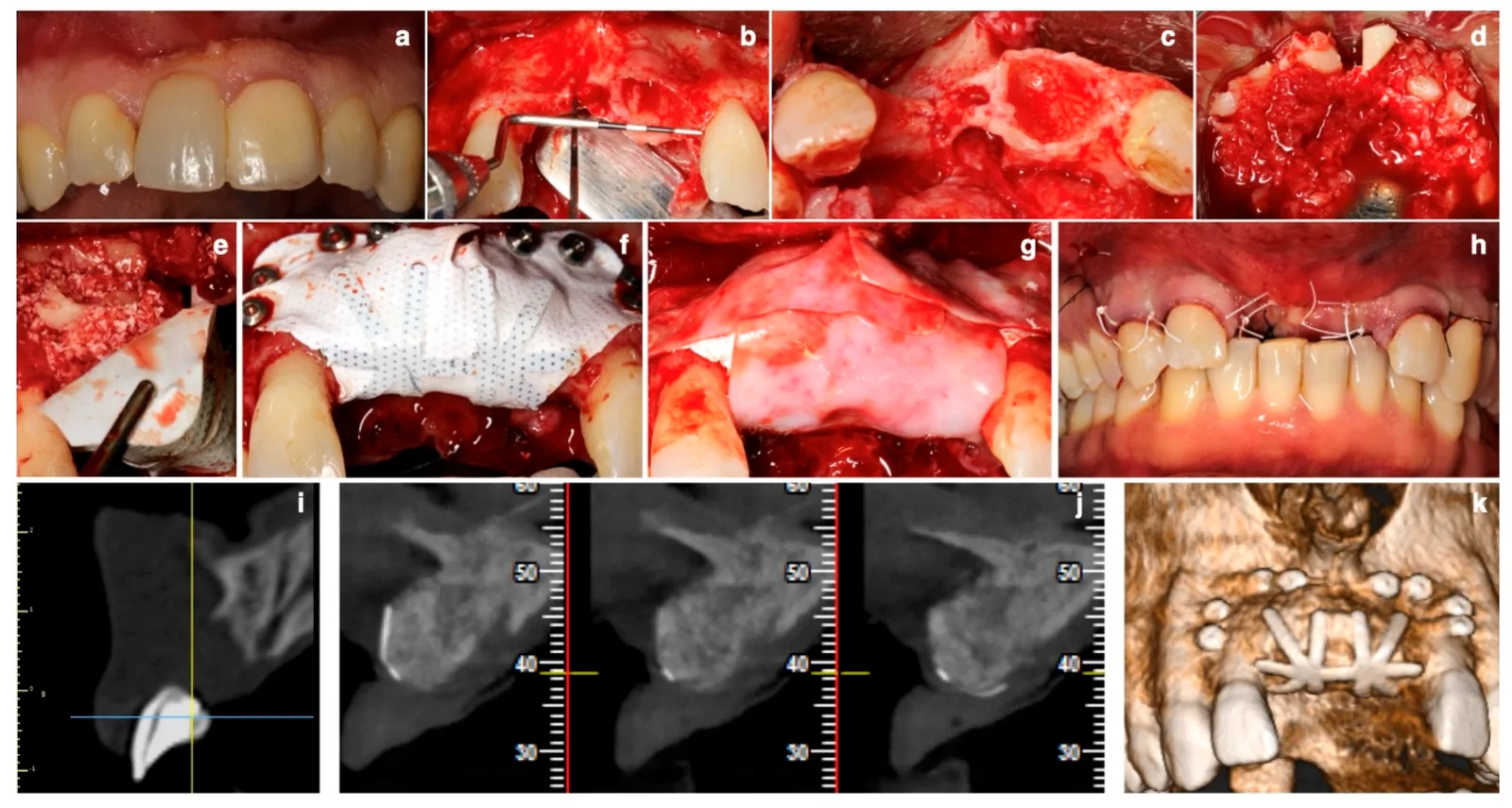
Representative clinical and radiographic sequence of vertical ridge augmentation using a titanium-reinforced dense PTFE (TiR-dPTFE) membrane in the anterior maxilla. (**a**) Frontal clinical view at baseline. (**b**) Measurement of vertical and horizontal defect dimensions after elevation of a full-thickness mucoperiosteal flap. (**c**) Occlusal view of the exposed defect morphology after flap reflection. (**d**) Autogenous bone cores and cortical chips harvested from the mandibular ramus. (**e**) Placement of the particulate graft mixture (autogenous bone and anorganic bovine bone mineral, 60:40 ratio) and initial stabilization of the TiR-dPTFE membrane on the palatal aspect. (**f**) Membrane fixation on both palatal and buccal aspects with titanium pins to ensure space maintenance. (**g**) Coverage of the TiR-dPTFE membrane with a native collagen membrane prior to closure. (**h**) Labial view showing tension-free primary flap closure at the end of surgery. (**i**) Preoperative cone-beam computed tomography (CBCT) cross-section. (**j**) CBCT cross-section at 9 months showing regenerated ridge volume. (**k**) Three-dimensional CBCT reconstruction at 9 months demonstrating augmented ridge morphology.

**Figure 3:**
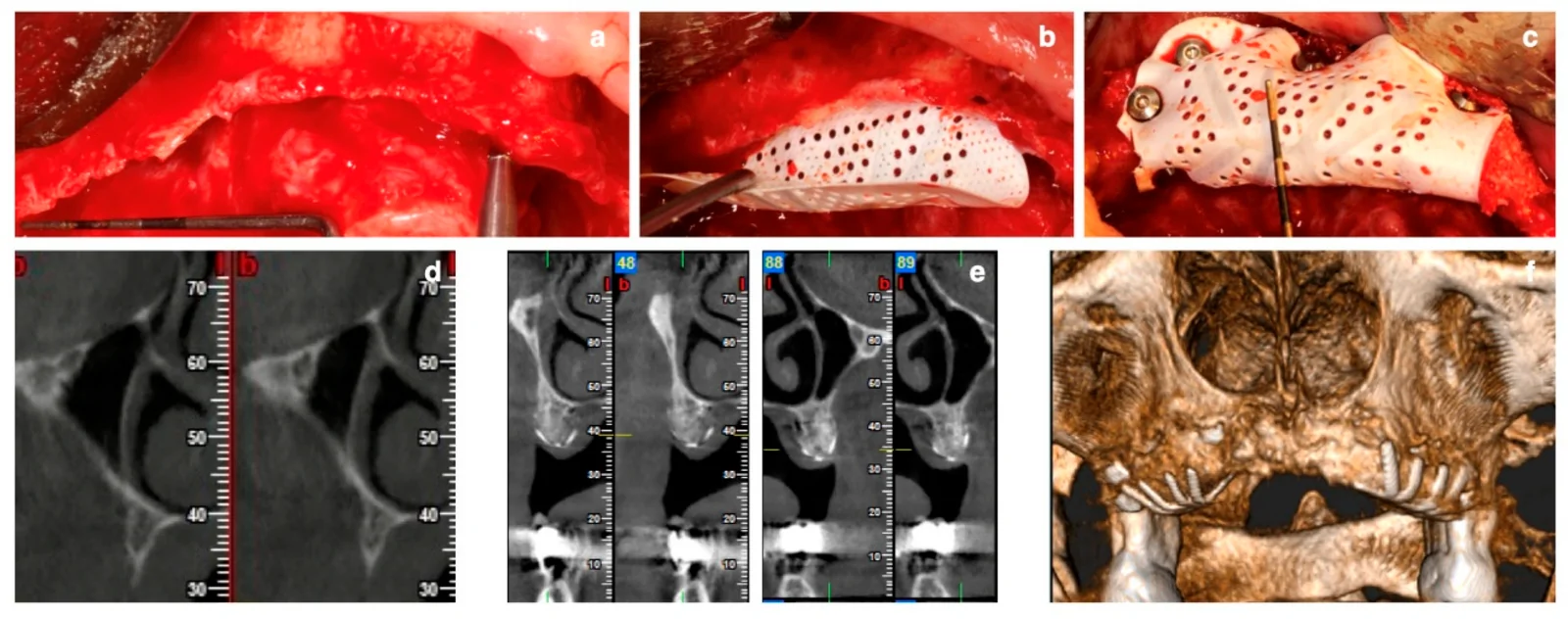
Representative clinical and radiographic sequence of vertical ridge augmentation using a reinforced PTFE mesh (RPM) membrane in the right hemi-maxilla. (**a**) Measurement of vertical and horizontal defect dimensions after elevation of a full-thickness mucoperiosteal flap at baseline. (**b**) Initial stabilization of the RPM membrane on the palatal aspect following placement of the particulate graft. (**c**) Fixation of the RPM membrane on both buccal and palatal aspects with titanium pins over a graft mixture composed of autogenous bone and anorganic bovine bone mineral (60:40 ratio). (**d**) Preoperative cone-beam computed tomography (CBCT) cross-section. (**e**) CBCT cross-section at 9 months showing regenerated ridge volume. (**f**) Three-dimensional CBCT reconstruction at 9 months demonstrating augmented ridge morphology.

Autogenous bone chips, harvested with a bone scraper (Safescrapper^®^, Meta, Italy), were mixed with deproteinized bovine bone matrix (Bio-Oss^®^, Geistlich Pharma, Wolhusen, Switzerland) in a 60:40 ratio. In the TiR-dPTFE group, the membrane was shaped to fully cover the graft, positioned 2 mm from adjacent native bone, and fixed with titanium pins (Master-Pin^®^, Meisinger, Neuss, Germany), then overlaid with a resorbable collagen membrane (Creos^®^ xenoprotect, Nobel Biocare, Switzerland) (Figure 2). In the RPM group, the reinforced PTFE membrane was similarly adapted, fixed with titanium pins, and covered with the same collagen membrane (Figure 3).

Tension-free primary closure was achieved using a double-layer PTFE suture technique (Cytoplast^®^, Osteogenics, Lubbock, TX, USA): a horizontal mattress suture, placed 5 mm from the incision, promoted flap eversion, and interrupted sutures provided additional stabilization and secure adaptation.

After the surgery, patients followed a comprehensive postoperative medical protocol. This included a regimen of antibiotics (1 g amoxicillin/clavulanic acid every 12 h for 7 days) and mouth rinsing (0.2% chlorhexidine mouthwash for 2 min, three times a day for 14 days). Postoperative instructions included ice therapy for 72 h, a soft and cold diet for 14 days, avoidance of rinsing and spitting for 72 h, refraining from any stress or physical exertion at the surgical site for 20 days, and abstaining from chewing in the area for 6 months. The sutures were removed 14–21 days after the surgery.

Throughout the healing period, removable partial dentures were not used to avoid interference with surgical site recovery. Patients underwent evaluations at 14 days (for suture removal), 30 days, 3 months, and 6 months following GBR to monitor for any adverse events.

A second CBCT scan was performed at 9 months (T1) to assess bone augmentation and to plan the implant surgery using DTX Studio Implant^®^ software (Nobel Biocare AB, Gothenburg, Sweden). Implants were planned 1 mm subcrestally and aligned with the prosthetic restoration plan.

**Figure 4:**
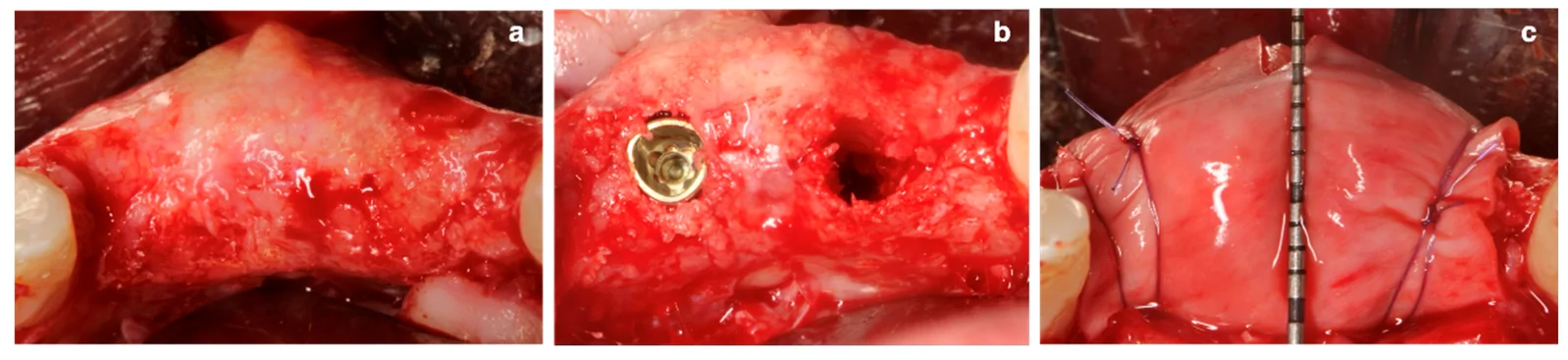
Re-entry surgical procedure at 9 months following vertical ridge augmentation with TiR-dPTFE membrane. (**a**) Re-opening of the surgical site after 9 months of healing and removal of the titanium-reinforced dense PTFE (TiR-dPTFE) membrane, showing the regenerated ridge. (**b**) Implant placement in prosthetically guided positions within the augmented bone volume. (**c**) Protective secondary grafting performed using a particulate mixture of anorganic bovine bone mineral (ABBM) and autogenous bone chips (70:30 ratio), covered with a native collagen membrane and stabilized with resorbable vertical mattress sutures.

A minimal mucoperiosteal flap was carefully elevated to expose the fixation pins and investigational device, facilitating their removal and enabling implant placement. To support the newly augmented bone, pseudo-periosteum was removed, and a secondary graft composed of DBBM and autogenous bone chips (70:30 ratio) was applied, covered with a native collagen membrane (Creos^®^ xenoprotect, Nobel Biocare, Zurich, Switzerland), and secured with resorbable vertical mattress sutures (Figure 4).

After 3 months, following confirmation of osseointegration, the implants were uncovered, and healing abutments were placed. At this stage, a new CBCT scan (T2) was also performed. Peri-implant soft tissue management was conducted using either a connective tissue graft or a strip gingival graft, as previously described.

#### 2.4.3. Histological Analyses

Biopsy specimens suitable for histological evaluation were obtained from 4 sites in the TiR-dPTFE group and 4 sites in the RPM group, when clinically feasible at the time of implant placement. Biopsies were collected using a trephine drill at T1, labeled, and immediately fixed in 4% formaldehyde. After decalcification in EDTA, the bone cylinder was carefully removed from the drill and divided into two portions. One portion was embedded in paraffin for conventional histological analysis, while the other was embedded in LR White resin for specialized microscopic evaluation. Histological sections were prepared from both embedded samples and stained with hematoxylin and eosin (H&E) to examine cytoplasmic, nuclear, and extracellular matrix structures under light microscopy.

#### 2.4.4. Measurements

A site-based analytical approach was adopted, as previously described in vertical ridge augmentation studies, since individual patients could present multiple independent implant sites exhibiting localized vertical defects suitable for treatment. Each defect site was analyzed separately on matched CBCT cross-sections using standardized anatomical landmarks, following established protocols. All radiographic measurements were performed by a single calibrated examiner (MP), blinded to treatment allocation and independent from the planning clinician (F.F.), to ensure objective and unbiased assessment.

### 2.5. Main Outcome Variable

The primary outcomes were VBG and horizontal bone gain (HBG), expressed in millimeters (mm). For each implant site, vertical bone defect (VBD) and horizontal bone defect (HBD) dimensions were first measured at baseline (T0) from cross-sectional views of the preoperative CBCT, using the planned bone volume as the reference (Figure 1). Postoperative CBCT scans at T1 and T2 were then analyzed in the corresponding cross-sectional planes, following the same protocol to determine VBG and HBG.

All measurements were performed by a calibrated examiner investigator (MP), blinded to the membrane type and treatment group, using ImageJ software, version 1.54 (Wayne Rasband. National Institute of Health, Bethesda, MD, USA). To test intraexaminer reliability, an assessment of 36 randomly selected measurements was repeated after 4 weeks. The intraclass correlation coefficient (ICC) was 0.93 (95% CI: 0.86 to 0.97; *p* < 0.001), indicating excellent absolute agreement.

### 2.6. Secondary Outcomes Variables

Adverse events: adverse events were classified as major or minor. Major events were defined as any complication requiring additional surgical intervention, premature membrane removal, or antibiotic therapy beyond the standard postoperative regimen. Minor events, including transient swelling or mild discomfort, were systematically recorded based on patient-reported outcomes and clinical examinations.

Histological measurements: Cytoplasmic, nuclear, and extracellular matrix structures were assessed to evaluate tissue response and integration.

### 2.7. Statistical Analysis

Analyses were conducted using the SPSS version 29 statistical package (SPSS Inc., Chicago, IL, USA). Categorical outcomes are presented as absolute and relative frequencies. The normality of scale variables was explored using the Shapiro–Wilk test and through visual analysis of the P-P plot and box plot. The interquartile range (IQR) and median were calculated for variables with non-normal distributions, while the mean and standard deviation (SD) were calculated for variables with a normal distribution. Two levels of analyses were considered: patient and site.

At the patient level, simple binary logistic regression was used to assess the homogeneity of study groups for both continuous and categorical variables. At the site level, univariate generalized linear mixed-effects models (GLMMs) with a linear response were fitted to examine the association between each covariate and the radiographic outcomes, accounting for clustering of multiple sites within the same patient. Crude mean differences (MDs) with corresponding 95% confidence intervals (95% CIs) were calculated for each covariate.

A multivariate analysis was performed using GLMMs with a forced-entry approach. Each model included treatment group (RPM and TiR-dPTFE), all covariates with a *p* value < 0.30 in the bivariate analysis, and the interaction between group and time as fixed-effect predictors. Adjusted MDs (MD_a_) with 95% confidence intervals were derived from the *t*-statistic, with statistical significance set at *p* < 0.05 and Bonferroni correction applied for multiple comparisons. Model assumptions were verified prior to interpretation.

## 3. Results

### 3.1. Demographics

A total of 46 vertical bone defect sites in 14 participants, whose ages ranged from 29 to 68 years, were treated with vertical and horizontal augmentation using GBR with either TiR-dPTFE or RPM membranes. Kennedy class II, class III and class IV partially edentulous patients were treated. Baseline characteristics were comparable across groups. The main patient- and site-level features, stratified by treatment group, are presented in Table 1, while Table 2 summarizes baseline defect morphology and distribution according to jaw, location and membrane type.

Because a standardized protective secondary augmentation (70:30 ABBM/autogenous with collagen membrane coverage) was performed at implant placement at all sites, the 12-month CBCT measurements reflect the combined effect of the primary membrane-based augmentation and the secondary grafting procedure rather than the primary barrier device alone.

### 3.2. Clinical Outcomes at 9 and 12 Months

Throughout the postoperative follow-up, from the first week to nine months after GBR with TiR-dPTFE or RPM membranes, all cases progressed without major complications. All patients experienced the expected and self-limiting postoperative responses, including mild-to-moderate discomfort and localized swelling during the first postoperative week. These symptoms resolved spontaneously within 7–10 days under standard care, with no additional interventions required. Importantly, the nature, intensity, and duration of these transient events were consistent across both treatment groups, with no clinically relevant differences observed. Throughout the follow-up period, no cases of membrane exposure, infection, or wound dehiscence occurred at any surgical site.

At nine months, all participants underwent a re-entry procedure for implant placement combined with simultaneous protective guided bone regeneration (secondary mini-graft), allowing implant placement in prosthetically driven positions at all treated sites. Adequate primary stability was achieved in each case. No adverse events were observed up to the 12-month evaluation prior to implant uncovering, and all implants remained stable throughout the healing phase and at the time of prosthetic loading.

Healing of both hard and soft tissues was uneventful at all surgical sites. Significant bone regeneration was evident at nine months, with the regenerated volume remaining stable at 12 months. All treated sites demonstrated substantial vertical and horizontal bone gains, confirming the effectiveness of both treatment protocols in achieving predictable tissue regeneration.

### 3.3. Radiographic Outcomes at 9 and 12 Months

**Figure 5:**
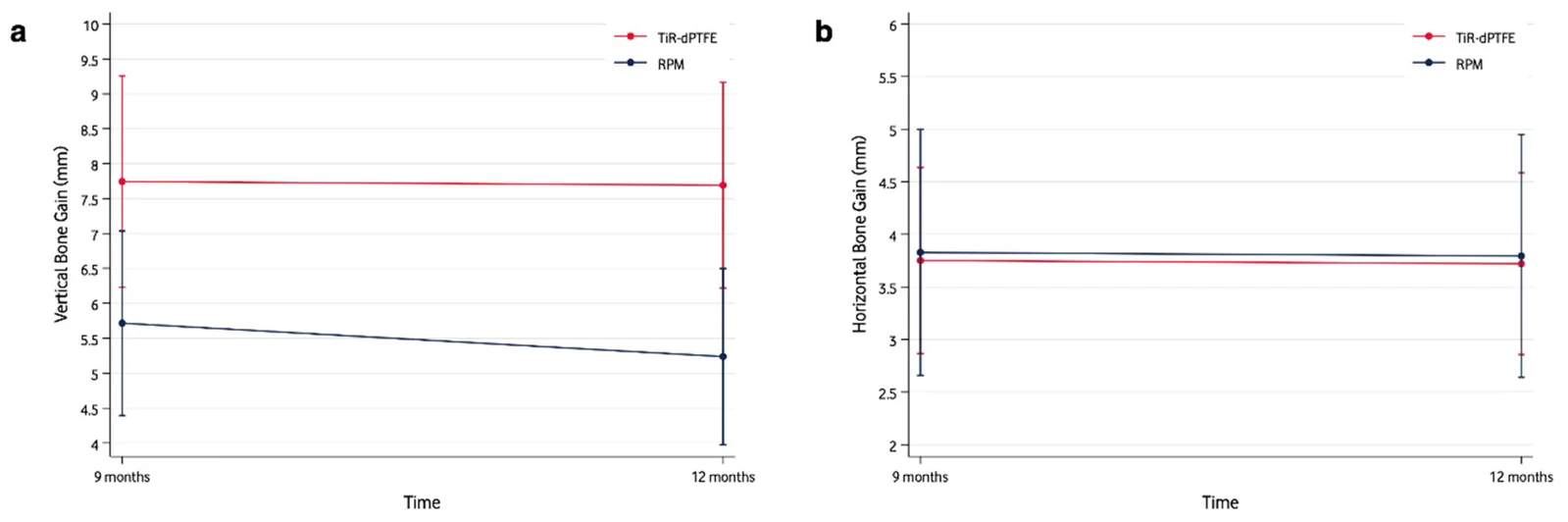
Changes in vertical bone gain (VBG) (**a**) and horizontal bone gain (HBG) (**b**) between 9 and 12 months following vertical ridge augmentation with titanium-reinforced dense PTFE (TiR-dPTFE) and reinforced PTFE mesh (RPM) membranes. The *y*-axis in panel A is truncated to improve visualization of between-group differences.

At 9 months, both groups showed significant VBG and HBG (both *p* < 0.001). TiR-dPTFE achieved greater VBG than RPM (MD_a_, 2.03 mm; 95% CI, 0.04 to 4.01; *p* = 0.045), whereas HBG did not differ between groups (MD_a_, −0.08 mm; 95% CI, −1.58 to 1.42; *p* = 0.918) (Table 3; Figure 5).

At 12 months, the VBG advantage for TiR-dPTFE persisted (MD_a_, 2.45 mm; 95% CI, 0.55 to 4.36; *p* = 0.012), while HBG remained comparable (MD_a_, −0.07 mm; 95% CI, −1.58 to 1.42; *p* = 0.922) (Table 3; Figure 5).

When comparing the two time points, TiR-dPTFE showed no significant change in VBG (MD_a_, −0.05 mm; 95% CI, −0.14 to 0.04; *p* = 0.255) or HBG (MD_a_, −0.03 mm; 95% CI, −0.07 to 0.01; *p* = 0.102). In contrast, RPM exhibited a significant reduction in VBG (MD_a_, −0.48 mm; 95% CI, −0.64 to −0.31; *p* < 0.001) with stable HBG (MD_a_, −0.03 mm; 95% CI, −0.08 to 0.01; *p* = 0.152) (Table 3; Figure 5).

### 3.4. Histological Outcomes at 9 Months

In a subset of representative cases, bone biopsies were harvested in a crestal position at the 9-month re-entry and processed for descriptive histological assessment. Quantitative comparison between groups was not pursued due to the limited and uneven number of specimens per membrane type, which would preclude a statistically meaningful analysis. The primary objective of histology was therefore to confirm bone vitality, graft integration, and the quality of the regenerated tissue.

**Figure 6:**
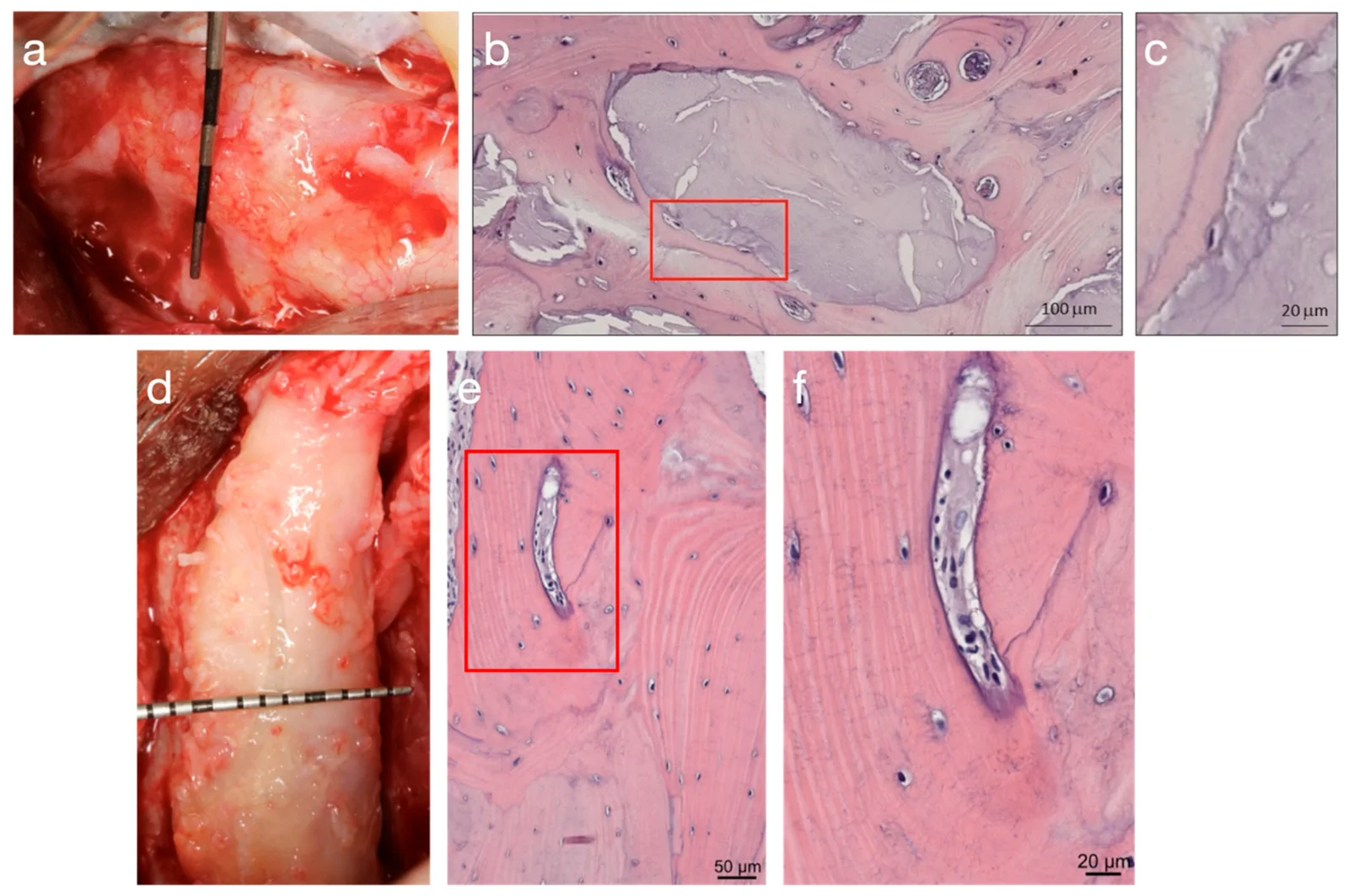
Representative clinical view and representative histological sections of regenerated bone at 9 months following vertical ridge augmentation with titanium-reinforced dense PTFE (TiR-dPTFE) (**a**–**c**) and reinforced PTFE mesh (RPM) (**d**–**f**). Histological sections show lamellar bone containing viable osteocytes, residual graft particles integrated within the mineralized matrix, bone marrow-like spaces, and signs of ongoing remodeling activity in both groups. Red boxes indicate magnified inset areas shown in the corresponding panels.

Critical assessment revealed no evidence of adverse tissue reactions, such as fibrosis or necrosis. Histological evaluation showed mature bone formation within the regenerated area in both groups, as illustrated by the clinical photographs (Figure 6a,d). Grafted material particles were incorporated within the newly formed bone matrix (Figure 6b), which exhibited lamellar organization, with viable osteocytes embedded in the calcified tissue. A direct interface was observed between residual graft particles and regenerated bone. Signs of ongoing remodeling activity were identifiable at the periphery of the grafted regions (Figure 6c). Bone marrow-like spaces were present within the lamellar bone (Figure 6b). No histological signs of inflammation were observed, and only limited soft-tissue components were present.

## 4. Discussion

The principle of guided bone regeneration (GBR) is to promote bone healing by preventing the invasion of rapidly proliferating fibrous tissue that may interfere with osteogenesis. By protecting the surgical site and maintaining a secluded environment for clot stabilization and osteogenic cell migration, GBR preserves the regenerative potential of surrounding tissues and enables predictable reconstruction of both vertical and horizontal ridge defects [25,26]. In this context, titanium-reinforced non-resorbable PTFE membranes are widely used in vertical ridge augmentation because of their ability to maintain space, stabilize particulate graft material, and provide effective soft-tissue exclusion during healing [9,10]. Clinical studies and randomized trials have consistently demonstrated substantial vertical bone gain using titanium-reinforced PTFE barriers combined with autogenous bone and xenograft mixtures, with outcomes comparable to those obtained using titanium mesh-based approaches [11,12,17,27]. Nevertheless, barrier configuration—including occlusive versus perforated designs—may influence vascularization patterns, graft protection, and early remodeling dynamics, which remain relevant determinants of dimensional stability after augmentation. Within this framework, differences between occlusive titanium-reinforced d-PTFE membranes and macroporous reinforced PTFE mesh configurations may influence the balance between space maintenance and periosteal vascular contribution during healing, potentially affecting the dimensional stability of regenerated bone following vertical ridge augmentation.

TiR-dPTFE membranes, used in combination with autogenous bone and ABBM, represent a widely established non-resorbable barrier strategy for vertical bone augmentation due to their higher structural rigidity and predictable space-maintaining capacity [9,28]. In contrast, RPMs incorporate calibrated microporosities intended to promote limited periosteal vascular contribution through direct interaction between the periosteum and the graft compartment while preserving the mechanical stability required for guided bone regeneration [27,29]. This hybrid configuration has been proposed to support graft maturation and soft-tissue integration without compromising barrier function.

Within the exploratory framework of this prospective comparative case series, the present findings should be interpreted primarily as hypothesis-generating observations rather than confirmatory evidence regarding the comparative performance of the two barrier configurations.

In the present study, TiR-dPTFE achieved greater VBG than RPM at both 9 months and 12 months, with stable values over time, whereas RPM demonstrated a modest but significant reduction between observation periods. These VBG magnitudes are consistent with previous reports on non-resorbable membranes and slightly exceed earlier TiR-dPTFE series using a 50:50 autogenous/ABBM mixture [9,11,12,21]. Although statistically significant, the mean reduction of approximately 0.5 mm observed in the RPM group represents a relatively small proportion of the total regenerated vertical height and remained compatible with implant placement in prosthetically driven positions at re-entry. In this context, the observed change is consistent with early remodeling phenomena commonly reported after vertical ridge augmentation procedures rather than indicative of clinically relevant loss of augmentation volume.

The greater VBG observed with TiR-dPTFE may be related to its greater resistance to graft compression and enhanced soft-tissue exclusion, both of which are critical determinants of regenerative predictability in vertical augmentation procedures. Conversely, the partial remodeling observed in the RPM group may reflect the biological consequences of its perforated architecture, which, while potentially facilitating periosteal vascular contribution, may also permit a greater degree of early graft adaptation during healing. However, causality cannot be established within the limits of the present study. Interpretation of the 12-month dimensional outcomes should consider that a standardized protective secondary augmentation (70:30 ABBM/autogenous with collagen membrane coverage) was performed at implant placement at all sites in the 9-month re-entry procedure; therefore, dimensional measurements obtained at 12 months reflect the combined effect of the primary membrane-based augmentation and the secondary grafting procedure rather than the barrier device alone, which limits attribution of late dimensional stability exclusively to membrane configuration.

Further, and although baseline VBD did not differ statistically between groups (*p* = 0.228), the numerically greater defect depth observed in the TiR-dPTFE group may have influenced the magnitude of vertical bone gain, potentially reflecting regression-to-the-mean effects. Given the limited number of patients included, baseline VBD was not incorporated as a covariate in the mixed-effects models to preserve model stability; consequently, the potential influence of baseline defect configuration on between-group differences cannot be excluded and represents an additional source of residual confounding.

In contrast to the vertical dimension, horizontal bone gain (HBG) remained comparable between groups at both time points, suggesting that width augmentation may be less dependent on barrier rigidity and may be similarly achievable with either device configuration within the limits of the present dataset. This observation is consistent with the established concept that vertical augmentation outcomes are more sensitive to mechanical stability and compartment protection than horizontal reconstruction procedures [30].

Although the primary endpoints of the present investigation were linear (VBG/HBG), their direction and magnitude are concordant with recent studies that quantified volumetric changes following vertical ridge augmentation using reinforced PTFE devices under standardized CBCT registration protocols [17,27,31]. In those reports, reconstructed sites generally demonstrate substantial net volumetric gains followed by partial remodeling/contraction over time, patterns that align with the present findings of sustained vertical stability in TiR-dPTFE and moderate vertical reduction in RPM [17,27,31]. These observations are further supported by a recent systematic review and network meta-analysis [25], which highlighted that early remodeling and partial contraction represent common biological features of vertical ridge augmentation even in randomized clinical settings.

Histologically, the two membranes demonstrated comparable patterns of tissue maturation at 9 months, characterized by lamellar bone containing viable osteocytes, residual graft particles integrated within the mineralized matrix, and clear evidence of ongoing remodeling activity. These findings are consistent with previous reports describing similar healing patterns following vertical augmentation with non-resorbable barrier devices [9,28,32]. Newly formed bone appeared partially immature in both groups at re-entry, supporting the concept that remodeling processes remain active at 9 months despite clinically adequate regeneration for implant placement [25,32]. Within this context, the comparable histological maturation observed between groups suggests that barrier configurations may have influenced dimensional stability more than the biological characteristics detectable at the time point evaluated, although quantitative histomorphometric confirmation was not available.

In vertical ridge augmentation, space-maintaining, form-stable devices (e.g., TiR-dPTFE and meshes) offer advantages over block grafting by reducing morbidity and operative complexity; however, complications such as flap dehiscence, membrane exposure, and infection remain relevant. Reported rates vary by material, with dPTFE membranes around 7% [32] and RPM closer to 3% [12]. This difference may reflect greater structural stability and tissue support with RPM, potentially improving soft-tissue management and reducing exposure risk in complex cases.

In this cohort, secondary augmentation was performed as part of a planned contouring strategy rather than as a response to insufficient regeneration. This approach aims to refine the crestal profile and enhance the stability of the regenerated volume before implant placement. Evidence supports this staged protocol, as delayed relining with particulate grafts and a resorbable membrane has been shown to improve dimensional maintenance following vertical augmentation procedures [33]. Within these parameters, secondary augmentation should be viewed as a complementary step to optimize architecture rather than an indication of primary regenerative failure.

In the present series, no major complications occurred in either group, supporting the clinical safety of both TiR-dPTFE and RPM in GBR. This favorable outcome may be partly related to the fact that all procedures were performed by a single surgeon with extensive experience in vertical ridge augmentation using non-resorbable barriers. However, the small sample size, single-center setting, and case-series design must be acknowledged, as these factors reduce the likelihood of capturing infrequent adverse events. Indeed, recent evidence indicates that complication rates, particularly membrane exposure, remain a common challenge across vertical augmentation techniques [34].

The exclusion of smokers likely contributed to the favorable outcomes observed. Smoking adversely affects every biological prerequisite for predictable guided bone regeneration, including vascularity, cellular recruitment, and wound stability. Nicotine causes vasoconstriction, carbon monoxide reduces tissue oxygenation, and smoke toxins impair neutrophil and fibroblast function; in addition, reduced salivary immunity and shifts in the oral microbiota increase susceptibility to infection. Clinically, these mechanisms lead to higher rates of early wound dehiscence, membrane exposure, graft contamination, and partial or complete graft loss. These risks are especially pronounced in vertical or extensive horizontal augmentation, where soft tissue tension and reduced perfusion already challenge healing, making smoking a high-impact modifiable factor. Across studies, both light smoking (under 5 to 10 cigarettes per day) and heavy smoking (over 20 cigarettes per day) are associated with more early wound complications and less net bone gain, although absolute rates vary depending on defect characteristics, flap design, membrane type, and surgeon experience [35].

Several limitations of this study should be acknowledged. First, the investigation was designed as a prospective comparative case series without randomization (14 patients; 46 implant sites), which limits causal inference and reduces statistical power for subgroup or interaction analyses. Although clustering was modeled using generalized linear mixed-effects models, the modest number of patients increases the risk of model overfitting and may leave residual patient-level confounding and imprecise effect estimates. Because multiple implant sites were nested within the same patients, the effective number of independent observational units corresponds to the number of patients rather than the total number of sites; this aspect should therefore be considered when interpreting between-group comparisons. The follow-up period of 9–12 months further constrains interpretation, as it precludes conclusions regarding long-term dimensional stability under functional loading. Despite excellent intra-examiner reliability (ICC = 0.93), CBCT-derived linear measurements remain susceptible to segmentation and landmarking variability. Additionally, a volumetric assessment was not performed, as the study was prospectively designed to evaluate linear outcomes (VBG/HBG) and lacked the registration aids (e.g., bite indices, fiducial markers) required for accurate pre-/post-operative CBCT superimposition; performing a retrospective volumetric analysis under these conditions could introduce substantial alignment bias. Histological evaluation was available only for a limited subset at 9 months and was reported qualitatively, restricting robust biological comparisons between membranes. Future studies should include larger and more balanced samples, prospectively standardized volumetric endpoints, and quantitative histomorphometric analyses to strengthen biological interpretation and enhance cross-study comparability. The re-entry protocol also included a secondary protective GBR at implant placement (70:30 ABBM/autogenous) with collagen-membrane coverage, which may have influenced dimensional changes between time points and complicates attribution solely to TiR-dPTFE or RPM. Finally, and although implant placement in prosthetically driven positions was successfully achieved in all cases, with adequate primary stability and uneventful progression through the early healing and loading phases, the absence of systematically collected long-term implant survival and prosthetic outcome data limits the ability to fully assess the long-term clinical impact of the regenerative procedures.

Overall, recent clinical and randomized studies evaluating titanium-reinforced PTFE membranes and reinforced PTFE mesh configurations have consistently reported predictable vertical bone gain when these barriers are used in combination with mixtures of autogenous bone and xenograft [9,12,29]. In particular, prospective and randomized investigations comparing titanium-reinforced PTFE membranes with titanium mesh-based approaches have demonstrated comparable dimensional augmentation outcomes, supporting the role of reinforced PTFE barriers as reliable space-maintaining devices in vertical ridge reconstruction [11,29]. Within this context, titanium reinforcement appears to be an important determinant of mechanical stability during healing, contributing to effective space maintenance and protection of the grafted compartment. The magnitude of vertical bone gain observed in the present study is consistent with previously reported values for non-resorbable PTFE-based techniques.

However, our results additionally suggest differences in short-term dimensional stability between barrier configurations, with TiR-dPTFE maintaining vertical gain between 9 and 12 months, while RPM showed a modest reduction over the same interval. These observations are in agreement with recent evidence indicating that early remodeling and partial contraction may occur after vertical augmentation procedures, particularly when barrier permeability allows greater periosteal interaction with the grafted compartment [36,37,38]. In addition, experimental evidence suggests that mesh porosity itself may influence regenerative outcomes, with smaller pore sizes being associated with improved bone formation and dimensional stability, further supporting the relevance of barrier architecture in determining healing dynamics [14].

Taken together, these findings suggest that although the macroporous design of RPM may support vascular contribution to the grafted compartment, the enhanced compartment containment and space-maintaining properties of TiR-dPTFE may represent a relevant factor for achieving stable vertical augmentation during the early healing phase within the limits of the present dataset. These observations should be interpreted cautiously given the non-randomized allocation strategy and the influence of the standardized secondary augmentation procedure performed at re-entry. Together, these findings are consistent with the concept that reinforced PTFE membranes can support predictable vertical regeneration under controlled clinical conditions, while barrier architecture may influence early stability of the augmented volume.

TiR-PTFE membranes may be particularly advantageous when maximum space maintenance and occlusion are required, such as vertical ridge augmentation with high risk of collapse, whereas RPM may represent a suitable alternative in situations where enhanced vascular contribution and biologic integration are considered clinically relevant, including defects requiring greater periosteal interaction with the grafted compartment.

Future work may explore bioactive or hybrid PTFE-based membranes that enhance vascularization and support biologic integration, as well as patient-specific 3D-printed designs that improve defect adaptation and reduce exposure risk. These emerging strategies should be evaluated in adequately powered comparative clinical studies with standardized volumetric endpoints and longer follow-up periods to determine their long-term stability and clinical effectiveness.

## 5. Conclusions

Vertical ridge augmentation using titanium-reinforced dense PTFE (TiR-dPTFE) and reinforced PTFE mesh (RPM) membranes in combination with autogenous bone and xenograft was associated with clinically feasible implant-site reconstruction and predictable horizontal augmentation outcomes within the limits of this prospective comparative case series. TiR-dPTFE demonstrated greater vertical bone gain at both 9 and 12 months, with improved short-term dimensional stability, whereas RPM showed a modest reduction in vertical gain between observation periods. These findings suggest that barrier configuration may influence early remodeling behavior following vertical augmentation, potentially reflecting differences in space maintenance and graft compartment protection.

Given the non-randomized allocation strategy, limited patient sample, and the influence of the standardized secondary augmentation procedure performed at re-entry, these findings should be interpreted as exploratory and hypothesis-generating rather than confirmatory. Within this context, both devices may represent viable options for vertical ridge augmentation under controlled clinical conditions, although their relative performance should be confirmed in adequately powered randomized clinical studies with standardized volumetric assessment and longer follow-up under functional loading.

## Figures and Tables

**Table 1:** Main patient and site features stratified by group (TiR-dPTFE: Titanium-reinforced dense PTFE membrane group, RPM: Reinforced PTFE mesh group). VBD: Vertical bone defect; HBD: Horizontal bone defect.

	TiR-dPTFE	RPM	*p*-Value
Patients	7	7	
Gender			0.559
Female	6 (85.71%)	4 (57.14%)	
Male	1 (14.29%)	3 (42.86%)	
Age (years) (SD)	55.36 (7.71)	51.21 (12.78)	0.479
Sites	23	23	
Site position			0.946
Anterior	12 (52.17%)	13 (56.52%)	
Posterior	11 (47.83%)	10 (43.48%)	
Arch			0.650
Maxilla	18 (78.26%)	15 (65.22%)	
Mandible	5 (21.74%)	8 (34.78%)	
Baseline VBD (mm) (SD)	8.31 (4.30)	6.78 (4.16)	0.228
Baseline HBD (mm) (SD)	4.69 (2.84)	5.16 (2.75)	0.570

**Table 2:** Main patient and site features stratified by group (TiR-dPTFE: Titanium-reinforced dense PTFE membrane group, RPM: Reinforced PTFE mesh group). VBD: Mean vertical bone defect; HBD: Mean horizontal bone defect. VBG: Mean vertical bone gain; HBG: Mean horizontal bone gain.

Patient	Group	Age	Sex	Number of Sites	Location	Position	Baseline VBD (mm)	Baseline HBD (mm)	Final VBG (mm)	Final HBG (mm)
1	TiR-dPTFE	68	Male	2	Maxilla	Anterior/Posterior	12.50	1.20	8.00	2.25
2	RPM	52	Female	6	Maxilla	Anterior/Posterior	5.90	3.00	6.15	2.37
3	TiR-dPTFE	35	Female	2	Maxilla	Anterior	14.80	5.20	9.80	4.65
4	TiR-dPTFE	29	Female	5	Mandible	Anterior/Posterior	8.50	4.70	8.40	4.20
5	RPM	51	Female	2	Maxilla	Anterior	3.10	7.50	4.13	4.31
6	TiR-dPTFE	45	Female	2	Maxilla	Posterior	3.50	5.90	6.75	4.85
7	RPM	52	Female	3	Maxilla	Anterior	15.40	6.10	10.00	3.67
8	RPM	33	Male	2	Mandible	Posterior	3.10	1.40	2.40	1.70
9	TiR-dPTFE	57	Female	3	Maxilla	Posterior	3.10	7.40	3.77	5.87
10	RPM	34	Male	4	Maxilla	Anterior/Posterior	6.90	3.50	5.40	2.55
11	TiR-dPTFE	66	Female	3	Maxilla	Posterior	8.80	0.50	7.65	1.80
12	RPM	46	Female	4	Mandible	Anterior/Posterior	7.70	5.40	4.45	3.30
13	RPM	63	Male	2	Mandible	Posterior	5.40	9.30	5.13	4.93
14	TiR-dPTFE	40	Female	6	Maxilla	Anterior/Posterior	6.90	7.80	7.50	5.87

**Table 3:** Changes in vertical and horizontal bone gain (primary outcomes) registered in the two study groups. The decrease in values indicates bone loss over the course of the study (VBG: vertical bone gain; HBG: horizontal bone gain; TiR-dPTFE: Titanium-reinforced dense PTFE membrane group; RPM: Reinforced PTFE mesh group; *: statistically significant difference).

**VBG (mm)**	**TiR-dPTFE** **Mean (SD)**	**RPM** **Mean (SD)**	***p*-Value (Group)**	**Cohen’s d**
9 months	7.74 (3.65)	5.72 (3.19)	0.045 *	0.59
12 months	7.69 (3.55)	5.24 (3.04)	0.012 *	0.74
*p*-value (time)	0.255	<0.001 *		
**HBG (mm)**	**TiR-dPTFE** **Mean (SD)**	**RPM** **Mean (SD)**	***p*** **-Value (Group)**	**Cohen’s d**
9 months	3.75 (2.13)	3.83 (2.83)	0.918	−0.03
12 months	3.72 (2.09)	3.79 (2.79)	0.922	−0.03
*p*-value (time)	0.102	0.152		

## Data Availability

The original contributions presented in the study are included in the article/Supplementary Material, further inquiries can be directed to the corresponding authors.

## Supplementary Materials

The following supporting information can be downloaded at: https://www.mdpi.com/2079-4983/17/5/234/s1, Figure S1. Patient screening, enrolment, allocation, follow-up, and analysis flow chart.

## References

[1] Elgali I., Omar O., Dahlin C., Thomsen P. (2017). Guided bone regeneration: Materials and biological mechanisms revisited. Eur. J. Oral Sci..

[2] Jepsen S., Schwarz F., Cordaro L., Derks J., Hammerle C.H.F., Heitz-Mayfield L.J., Hernandez-Alfaro F., Meijer H.J.A., Naenni N., Ortiz-Vigon A. (2019). Regeneration of alveolar ridge defects. Consensus report of group 4 of the 15th European Workshop on Periodontology on Bone Regeneration. J. Clin. Periodontol..

[3] Mierzejewska Ż.A., Veselinović V., Trtić N., Marin S., Borys J., Antonowicz B. (2026). Advanced Biomaterials for Craniofacial Tissue Regeneration: From Fundamental Mechanism to Translational Applications—A Scoping Review. J. Funct. Biomater..

[4] Abtahi S., Chen X., Shahabi S., Nasiri N. (2023). Resorbable Membranes for Guided Bone Regeneration: Critical Features, Potentials, and Limitations. ACS Mater. Au.

[5] Ren Y., Fan L., Alkildani S., Liu L., Emmert S., Najman S., Rimashevskiy D., Schnettler R., Jung O., Xiong X. (2022). Barrier Membranes for Guided Bone Regeneration (GBR): A Focus on Recent Advances in Collagen Membranes. Int. J. Mol. Sci..

[6] Chiapasco M., Abati S., Romeo E., Vogel G. (1999). Clinical outcome of autogenous bone blocks or guided bone regeneration with e-PTFE membranes for the reconstruction of narrow edentulous ridges. Clin. Oral Implant. Res..

[7] Lima-Sánchez B., Baus-Domínguez M., Serrera-Figallo M.A., Torres-Lagares D. (2025). Advances in synthetic polymer membranes for guided bone regeneration in dental implants: A scoping review. J. Funct. Biomater..

[8] Carbonell J.M., Martin I.S., Santos A., Pujol A., Sanz-Moliner J.D., Nart J. (2014). High-density polytetrafluoroethylene membranes in guided bone and tissue regeneration procedures: A literature review. Int. J. Oral Maxillofac. Surg..

[9] Urban I.A., Lozada J.L., Jovanovic S.A., Nagursky H., Nagy K. (2014). Vertical ridge augmentation with titanium-reinforced, dense-PTFE membranes and a combination of particulated autogenous bone and anorganic bovine bone-derived mineral: A prospective case series in 19 patients. Int. J. Oral Maxillofac. Implant..

[10] Soldatos N., Garcia M., Umoh E., Irizarry A., Weltman R. (2022). Vertical Ridge Augmentation Around Dental Implants With the Use of a Dense PTFE Membrane to Correct Previously Failed Augmentations. Clin. Adv. Periodontics.

[11] Cucchi A., Vignudelli E., Napolitano A., Marchetti C., Corinaldesi G. (2017). Evaluation of complication rates and vertical bone gain after guided bone regeneration with non-resorbable membranes versus titanium meshes and resorbable membranes. A randomized clinical trial. Clin. Implant Dent. Relat. Res..

[12] Urban I.A., Saleh M.H.A., Ravida A., Forster A., Wang H.L., Barath Z. (2021). Vertical bone augmentation utilizing a titanium-reinforced PTFE mesh: A multi-variate analysis of influencing factors. Clin. Oral Implant. Res..

[13] Yousefi-Koma A.A., Amid R., Moscowchi A., Nokhbatolfoghahaei H., Kadkhodazadeh M. (2025). Accompanying Titanium Meshes and Titanium-Reinforced Membranes with Collagen Membranes in Vertical Alveolar Ridge Augmentations: A Systematic Review. J. Funct. Biomater..

[14] Mantovani R., Fernandes Y., Meza-Mauricio J., Reino D., Gonçalves L.S., Sousa L.G., Almeida A.L., Faveri M., Scombatti de Souza S. (2023). Influence of Different Porosities of Titanium Meshes on Bone Neoformation: Pre-Clinical Animal Study with Microtomographic and Histomorphometric Evaluation. J. Funct. Biomater..

[15] World Medical Association (2013). World Medical Association Declaration of Helsinki: Ethical principles for medical research involving human subjects. JAMA.

[16] Vandenbroucke J.P., Von Elm E., Altman D.G., Gøtzsche P.C., Mulrow C.D., Pocock S.J., Poole C., Schlesselman J.J., Egger M., Strobe Initiative (2014). Strengthening the Reporting of Observational Studies in Epidemiology (STROBE): Explanation and elaboration. Int. J. Surg..

[17] Cucchi A., Bettini S., Tedeschi L., Urban I., Franceschi D., Fiorino A., Corinaldesi G. (2024). Complication, vertical bone gain, volumetric changes after vertical ridge augmentation using customized reinforced PTFE mesh or Ti-mesh. A non-inferiority randomized clinical trial. Clin. Oral Implant. Res..

[18] Amaral Valladao C.A., Freitas Monteiro M., Joly J.C. (2020). Guided bone regeneration in staged vertical and horizontal bone augmentation using platelet-rich fibrin associated with bone grafts: A retrospective clinical study. Int. J. Implant Dent..

[19] Monje A., Monje F., Hernandez-Alfaro F., Gonzalez-Garcia R., Suarez-Lopez del Amo F., Galindo-Moreno P., Montanero-Fernandez J., Wang H.L. (2015). Horizontal Bone Augmentation Using Autogenous Block Grafts and Particulate Xenograft in the Severe Atrophic Maxillary Anterior Ridges: A Cone-Beam Computerized Tomography Case Series. J. Oral Implantol..

[20] Tinti C., Parma-Benfenati S. (1998). Vertical ridge augmentation: Surgical protocol and retrospective evaluation of 48 consecutively inserted implants. Int. J. Periodontics Restor. Dent..

[21] Urban I.A., Monje A., Nevins M., Nevins M.L., Lozada J.L., Wang H.L. (2016). Surgical Management of Significant Maxillary Anterior Vertical Ridge Defects. Int. J. Periodontics Restor. Dent..

[22] Urban I., Traxler H., Romero-Bustillos M., Farkasdi S., Bartee B., Baksa G., Avila-Ortiz G. (2018). Effectiveness of Two Different Lingual Flap Advancing Techniques for Vertical Bone Augmentation in the Posterior Mandible: A Comparative, Split-Mouth Cadaver Study. Int. J. Periodontics Restor. Dent..

[23] Noguera-Mutllo C., Traboulsi-Garet B., Camps-Font O., Manzanares-Cespedes M.C., Figueiredo R., Valmaseda-Castellon E. (2022). Comparison of two different lingual flap advancement techniques and vascular structure identification: A human cadaver study. Med. Oral Patol. Oral Cir. Bucal.

[24] Ronda M., Stacchi C. (2011). Management of a coronally advanced lingual flap in regenerative osseous surgery: A case series introducing a novel technique. Int. J. Periodontics Restor. Dent..

[25] Simion M., Jovanovic S.A., Tinti C., Benfenati S.P. (2001). Long-term evaluation of osseointegrated implants inserted at the time or after vertical ridge augmentation. A retrospective study on 123 implants with 1–5 year follow-up. Clin. Oral Implant. Res..

[26] Urban I.A., Montero E., Monje A., Sanz-Sanchez I. (2019). Effectiveness of vertical ridge augmentation interventions: A systematic review and meta-analysis. J. Clin. Periodontol..

[27] Urban I.A., Serroni M., Dias D.R., Barath Z., Forster A., Araujo T.G., Saleh M.H.A., Cucchi A., Ravida A. (2025). Impact of Collagen Membrane in Vertical Ridge Augmentation Using Ti-Reinforced PTFE Mesh: A Randomised Controlled Trial. J. Clin. Periodontol..

[28] Gallo P., Diaz-Baez D., Perdomo S., Aloise A.C., Tattan M., Saleh M.H.A., Pelegrine A.A., Ravida A., Wang H.L. (2022). Comparative analysis of two biomaterials mixed with autogenous bone graft for vertical ridge augmentation: A histomorphometric study in humans. Clin. Implant Dent. Relat. Res..

[29] Cucchi A., Bettini S., Ghensi P., Fiorino A., Corinaldesi G. (2023). Vertical ridge augmentation with Ti-reinforced dense polytetrafluoroethylene (d-PTFE) membranes or Ti-meshes and collagen membranes: 3-year results of a randomized clinical trial. Clin. Implant Dent. Relat. Res..

[30] Fu J.H., Choo H.J., Ong D.S., Kwek H. (2026). Long-term stability of horizontal bone augmentation at implant sites. Periodontology 2000.

[31] Felice P., Pistilli R., Pellegrino G., Bonifazi L., Tayeb S., Simion M., Barausse C. (2024). A randomised controlled trial comparing the effectiveness of guided bone regeneration with polytetrafluoroethylene titanium-reinforced membranes, CAD/CAM semi-occlusive titanium meshes and CAD/CAM occlusive titanium foils in partially atrophic arches. Int. J. Oral Implantol..

[32] Cucchi A., Sartori M., Parrilli A., Aldini N.N., Vignudelli E., Corinaldesi G. (2019). Histological and histomorphometric analysis of bone tissue after guided bone regeneration with non-resorbable membranes vs resorbable membranes and titanium mesh. Clin. Implant Dent. Relat. Res..

[33] De Stavola L., Tunkel J. (2013). A new approach to maintenance of regenerated autogenous bone volume: Delayed relining with xenograft and resorbable membrane. Int. J. Oral Maxillofac. Implant..

[34] Alotaibi F.F., Buti J., Rocchietta I., Mohamed Nazari N.S., Almujaydil R., D’Aiuto F. (2025). Premature Bone Resorption in Vertical Ridge Augmentation: A Systematic Review and Network Meta-Analysis of Randomised Clinical Trials. Clin. Oral Implant. Res..

[35] Jung R.E., Fenner N., Hammerle C.H., Zitzmann N.U. (2013). Long-term outcome of implants placed with guided bone regeneration (GBR) using resorbable and non-resorbable membranes after 12–14 years. Clin. Oral Implant. Res..

[36] Flynn R., Foschi F., Maloney B., Creavin G., Duncan H.F. (2024). The impact of bone grafting with/without barrier membrane placement on the outcome of apical surgery: A systematic review and meta-analysis. Int. Endod. J..

[37] Kunrath M.F., Magrin G.L., Zorzo C.S., Rigotto I., Aludden H., Dahlin C. (2025). Membranes for periodontal and bone regeneration: Everything you need to know. J. Periodontal Res..

[38] Donos N., Akcali A., Padhye N., Sculean A., Calciolari E. (2023). Bone regeneration in implant dentistry: Which are the factors affecting the clinical outcome?. Periodontology 2000.

