# Correction: Silva et al. Vertical and Horizontal Ridge Augmentation with Titanium-Reinforced Dense PTFE and Reinforced PTFE Mesh: A Prospective Comparative Case Series. *J. Funct. Biomater.* 2026, *17*, 234

**DOI:** 10.3390/jfb17100483

**Published:** 2026-09-23

**Authors:** Liliana Andrea Silva, Pedro Sousa Gomes, Maria Helena Fernandes, Marta García-García, Octavi Camps-Font

**Affiliations:** 1 Cirurgia Oral, Faculdade de Medicina Dentária, Universidade do Porto, 4200-393 Porto, Portugal; 2 Bone Lab, Faculdade de Medicina Dentária, Universidade do Porto, 4200-393 Porto, Portugal; mhfernandes@fmd.up.pt; 3 LAQV/REQUIMTE, Faculdade de Medicina Dentária, Universidade do Porto, 4200-393 Porto, Portugal; 4 Faculty of Medicine and Health Sciences, Universitat de Barcelona, 08036 Barcelona, Spain; martagarciagarcia@ub.edu (M.G.-G.); ocamps@ub.edu (O.C.-F.); 5 IDIBELL Research Institute, 08908 Barcelona, Spain

**Missing Informed Consent Statement**

In the original publication [1], the informed statement “Informed consent for participation was obtained from all subjects involved in the study.” is missing from the back-matter section. The authors state that the scientific conclusions are unaffected. This correction was approved by the Academic Editor. The original publication has also been updated.
