# Supplementary figures and images for: Vertical and Horizontal Ridge Augmentation with Titanium-Reinforced Dense PTFE and Reinforced PTFE Mesh: A Prospective Comparative Case Series
Source: J Funct Biomater. 2026 May 7;17(5):234. doi: 10.3390/jfb17050234 (PMC13207885; doi:10.3390/jfb17050234)

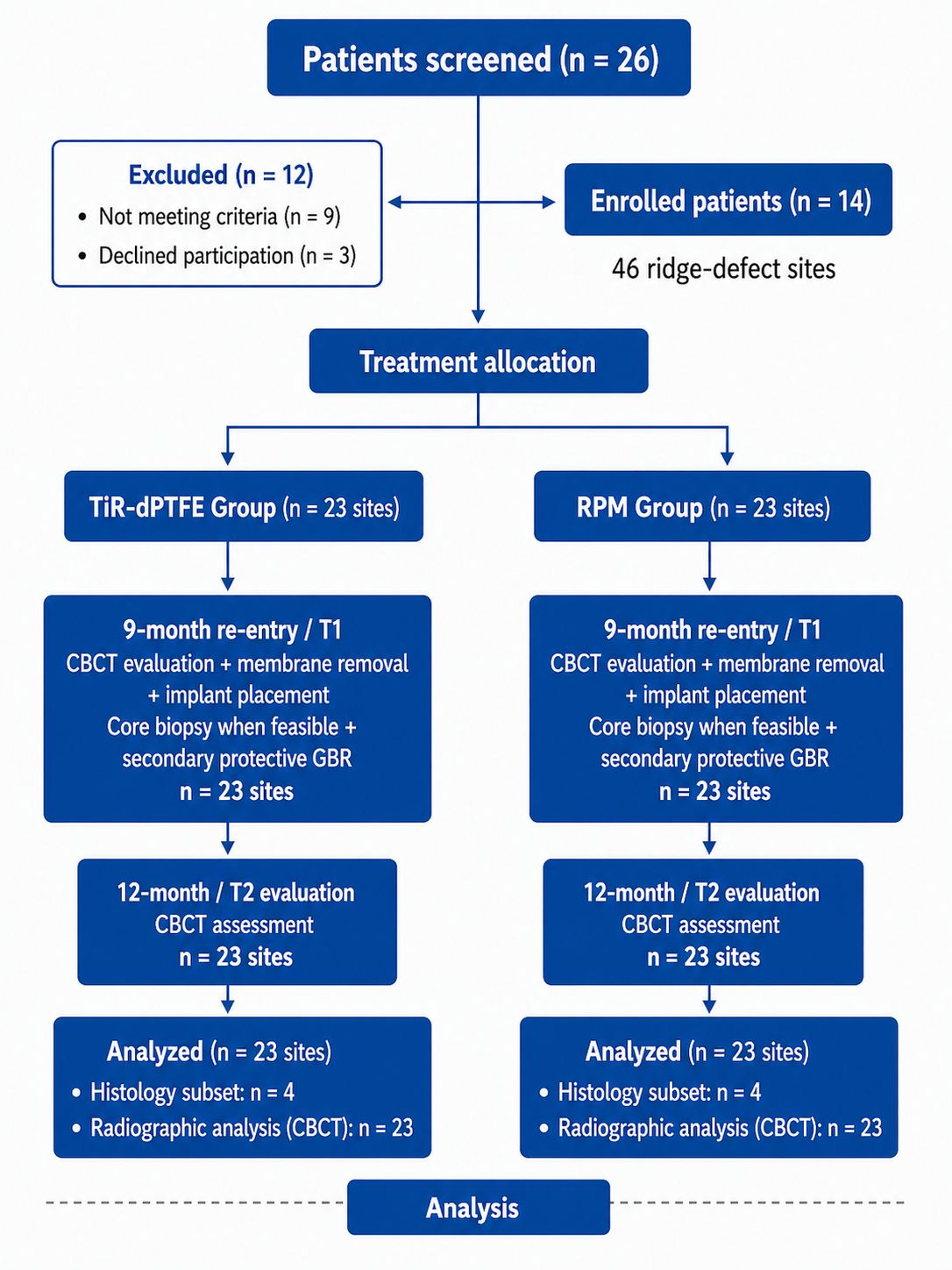

Supplement: Supplementary file 1 [file jfb-17-00234-s001.zip › jfb-4250840-supplementary.jpeg]
